# TissUUmaps: interactive visualization of large-scale spatial gene expression and tissue morphology data

**DOI:** 10.1093/bioinformatics/btaa541

**Published:** 2020-05-25

**Authors:** Leslie Solorzano, Gabriele Partel, Carolina Wählby

**Affiliations:** Department of Information Technology and SciLifeLab, Uppsala University, Uppsala 752 37, Sweden

## Abstract

**Motivation:**

Visual assessment of scanned tissue samples and associated molecular markers, such as gene expression, requires easy interactive inspection at multiple resolutions. This requires smart handling of image pyramids and efficient distribution of different types of data across several levels of detail.

**Results:**

We present TissUUmaps, enabling fast visualization and exploration of millions of data points overlaying a tissue sample. TissUUmaps can be used both as a web service or locally in any computer, and regions of interest as well as local statistics can be extracted and shared among users.

**Availability and implementation:**

TissUUmaps is available on github at github.com/wahlby-lab/TissUUmaps. Several demos and video tutorials are available at http://tissuumaps.research.it.uu.se/howto.html.

**Supplementary information:**

Supplementary data are available at *Bioinformatics* online.

## 1 Introduction

Histopathology refers to the examination of tissue samples through a microscope to study the manifestations of disease. Typically, tissue morphology is visualized by common stains such as hematoxylin and eosin (H&E). Today, novel techniques make it possible to also detect and visualize the molecular composition of the tissue with subcellular resolution, while preserving spatial information. Techniques like *in-situ* sequencing (ISS), MERFISH, osmFISH, seqFISH and STARmap explained in Strell *et al.* (2019), produce millions of spatially resolved data points describing transcript locations of hundreds to thousands of targeted genes. Technological advances allow for fast scanning with high resolution, resulting in gigapixel whole slide images (WSI) on one hand, and millions of data points on the other. Furthermore, tissue morphology, such as size, shape and neighborhood relations of cells, or more complex features defined by deep learning, can be quantified and presented as color-coded markers.

All these sources of information combined provide cues on manifestation of disease, and are pivotal for the discovery of new biomarkers relevant for clinical decision making and personalized medicine. As an analogy, one can combine geographic data (satellite and terrain images) with Global Positioning Data (spatial coordinates), where emerging patterns can be observed and new associations between information modalities can be studied.

To build open source software, certain considerations have to be taken into account. As explained in Marée (2019), a software can be made for running in the desktop (locally) or in the web. An example of a local WSI viewer is QuPath (Bankhead *et al.*, 2017) and examples of web viewers are OMERO.viewer (Allan *et al.*, 2012) and Cytomine (Marée *et al.*, 2016). They all require an extensive set of libraries to function, because they aim to cover a very broad range of digital pathology practices basically turning the computer into a full server with intricate interconnections between many different libraries.

TissUUmaps focuses on the information visualization aspect of data analysis where visual representations of data lead to better human cognition and interpretation. Apart from interaction, such as zooming, panning, changing of marker size and shape and switching between data from different imaging modalities, the user can also draw regions of interest and extract localized information to explore tissue heterogeneity. TissUUmaps is designed for three types of users; the user only wanting to view and explore data made available by someone else, the user that has own data and wants to use the standard functionality locally, and the advanced user that wants to provide it as a service and/or add new functionality. For the first category of users, no external installations are required because it only needs a web browser (e.g. Chrome, Firefox, Safari) which comes preinstalled in modern computers. To run locally, you only need to copy the TissUUmaps repository and transform the images to tiled format. To provide TissUUmaps as service, the flexibility of the open source framework makes it easy to deploy new, specialized tools, e.g. for viewing pairs of tissue slices simultaneously by instantiating synchronized viewers and selecting control points for aligning images of consecutive tissue slices. We have created a collection of video tutorials (tissuumaps.research.it.uu.se/howto.html) for a standard user and up to an advanced level on how to extend TissUUmaps.

Point data visualized by TissUUmaps are not limited to gene profiling. Working with the tiled WSI, individual cells can be segmented using tools such as CellProfiler (Kamentsky *et al.*, 2011) or Ilastik (Berg *et al.*, 2019), followed by automated extraction of a large number of different per-cell measurements, such as cell shape, area, perimeter, etc. After transformation to global coordinates, this type of information can be visualized as color-coded point data using TissUUmaps, quickly providing an overview of heterogeneity in tissue morphology.

## 2 Implementation, deployment and use

TissUUmaps is open source and created using standard web development tools, pure html, CSS and vanilla JavaScript. It uses a small number of libraries, as described below, making it easy to setup and maintain without containers.

In its base configuration, TissUUmaps organizes the data into image data and marker data. Image data are the WSI in a tiled format, and marker data are everything that can be overlaid on it, such as points representing gene expression, per-cell measurements, regions and annotations or any other spatially resolved data. This is reflected in the user interface where the corresponding tabs allow the user to navigate the data.

To display image data, we use OpenSeadragon (openseadragon.github.io) which can manage Deep Zoom pyramidal images (DZI) and additionally offers a scalable vector graphics (SVG) canvas on which point data can be drawn. Just like the map analogy where only the large cities are displayed at low resolution, TissUUmaps uses the statistical toolbox of the Data-Driven Documents (D3) library from Bostock *et al.* (2011), to process marker data prior to visualization. Only a representative subset of the data is presented as SVG elements on the canvas at low resolution viewing, while local details increase as the user zooms into a higher magnification of the sample, making interaction faster and less cluttered.

DZI can be created from a large number of different scanner formats thanks to the Open Microscopy Environment (Besson *et al.*, 2019) and the efforts of several open tools, specifically: VIPS (Martinez and Cupitt, 2005), OpenSlide and Bio-Formats (Linkert *et al.*, 2010).

The advanced user can add new buttons to call different functions, analysis and reporting capabilities. Additionally, extended functionality can be scripted in the browser console for filtering data or creating new visualizations using D3 and the TissUUmaps API.

To use TissUUmaps when offered as a service already setup, image data are browsed in the main viewer to the left, it can be zoomed in and panned, and the marker data can be loaded, drawn and explored using the options in the panel on the right.

To use TissUUmaps locally, it suffices to clone the repository, point the html file to the location of the images and then open the html file in any browser. Point source data should be provided in.csv file format to be loaded, and the images have to be converted to a tiled format. Detailed instructions for generating the tiled image, together with an example case and instructions, are available at our github repository.

To set up TissUUmaps as a service, we used an Apache web server. Other web servers, such as NGINX or a python http.server, would also work as TissUUmaps is a basic html file and does not need a special web framework.

## 3 Conclusion

**Fig. 1.**
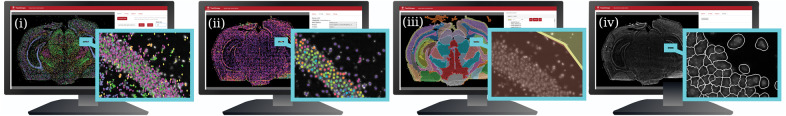
TissUUmaps can simultaneous display image data and point data, e.g. DAPI stained cell nuclei imaged using fluorescence microscopy combined with: (**i**) gene expression, (**ii**) cell morphology (measured by CellProfiler), (**iii**) manually or automatically drawn regions and (**iv**) cell outlines (segmented by CellProfiler). Multiple viewers can be instantiated and synchronized. TissUUmaps allows for operations in the image data through plugins, such as shaders, brightness and contrast modification

A case study for TissUUmaps is presented in Partel *et al.* (2019) where more than two million gene transcripts were decoded from a 97 gene panel ISS assay of a coronal section of mouse brain (Fig. 1i). The results can be interactively visualized and explored with TissUUmaps at the demo (tissuumaps.research.it.uu.se/demo/isseq.html and isseq2.html). Spatial anatomical compartments automatically defined in the same study can also be imported and visualized in TissUUmaps (Fig. 1iii), along with 16 different morphological features extracted with CellProfiler (Fig. 1ii), or overlaying and comparing with cellular morphology from the underlying nuclei stain (Fig. 1iv).

TissUUmaps also offers the possibility to visualize more complex features as spatial gene constellation learned by spage2vec (tissuumaps.research.it.uu.se/spage2vec) from Partel and Wählby (2020), representing different subcellular spatial domains involved in cellular differentiation. Another example is presented in Ström *et al.* (2020) where H&E slides of prostate biopsies are synchronized with a prediction map, displayed using WebGL shaders (tissuumaps.research.it.uu.se/STHLM3).

There are several visualization options for WSI but to our knowledge, none offer an easy and convenient way for visualizing millions of data points along with image data. Additionally, TissUUmaps can be used both as a web service or locally in any computer and does not have any prerequisites apart from a modern web browser.

## Supplementary Material

btaa541_Supplementary_Data
