# Supplementary material for: TissUUmaps: interactive visualization of large-scale spatial gene expression and tissue morphology data
Source: Bioinformatics. 2020 May 25;36(15):4363–5. doi: 10.1093/bioinformatics/btaa541 (PMC7520034; doi:10.1093/bioinformatics/btaa541)
Supplement: btaa541_Supplementary_Data [file btaa541_supplementary_data.pdf]

TissUUmaps

Supplementary data

Available demos **1**

Documentation **3**

For the user of an already setup TissUUmaps viewer 3

For setting up a local TissUUmaps viewer 4

Converting images to tiled formats 4

Pointing TissUUmaps to your image data 4

Additional information for those curious 5

For setting up TissUUmaps as a service 6

TissUUmaps API 8

Examples 9

Changing layer transparency/opacity 9

Making a histogram of the barcodes in a region 11

More examples 13

Available demos

TissUUmaps can be used to display many different kinds of point data. We have set up several demos where different tissue and gene expression and properties can be explored.

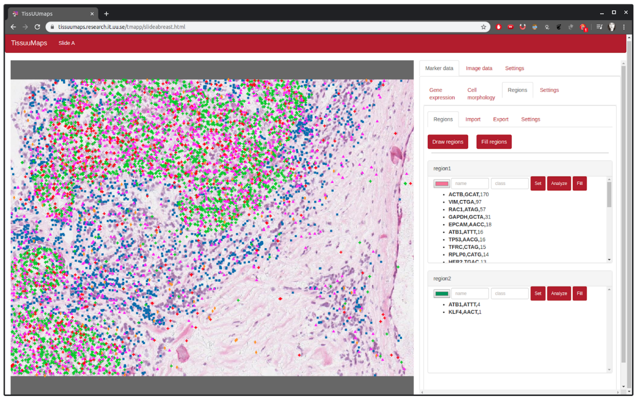

In situ sequencing in breast cancer slide

TissUUmaps demo:
https://tissuumaps.research.it.uu.se/demo/slideabreast.html

Paper:
Rongqin Ke et al.
In situ sequencing for RNA analysis in preserved tissue and cells
doi.org/10.1038/nmeth.2563

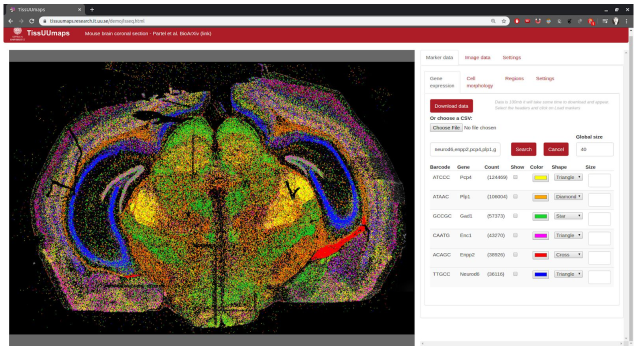

Mouse brain gene expression decoding Brain 1

TissUUmaps demo:
https://tissuumaps.research.it.uu.se/demo/isseq.html

Paper:
Partel et al.
Identification of spatial compartments in tissue from in situ sequencing data
doi.org/10.1101/765842

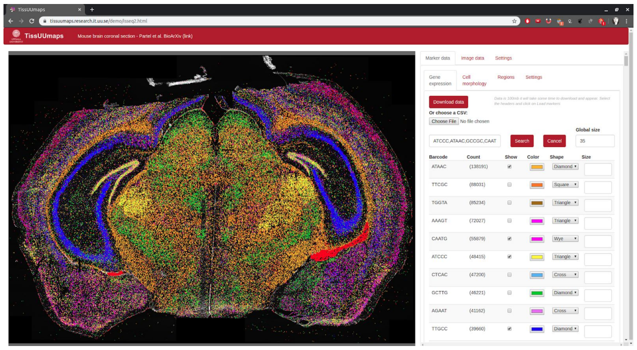

Mouse brain gene expression decoding Brain 2

TissUUmaps demo:
https://tissuumaps.research.it.uu.se/demo/isseq.html

Paper:
Partel et al.
Identification of spatial compartments in tissue from in situ sequencing data
doi.org/10.1101/765842

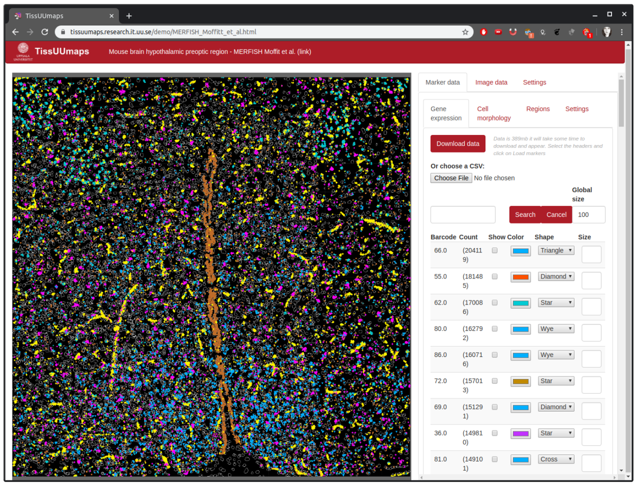

MERFISH mouse brain hypothalamic preoptic area TissUUmaps demo:
https://tissuumaps.research.it.uu.se/demo/MERFISH_Moffitt_et_al.html

Paper:
Moffitt et al
Molecular, spatial, and functional single-cell profiling of the hypothalamic preoptic region.
doi.org/10.1126/science.aau5324

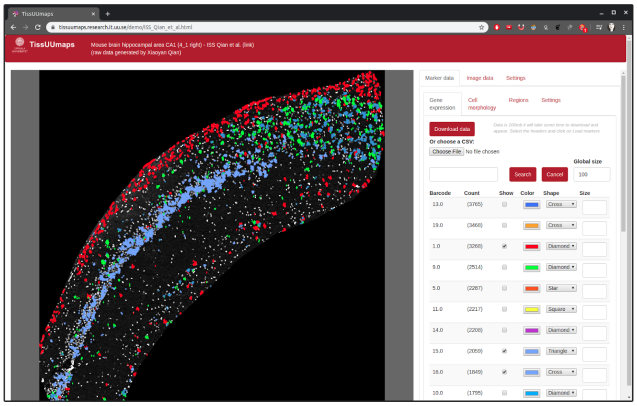

In situ sequencing mouse brain hippocampal area CA1

TissUUmaps demo:
https://tissuumaps.research.it.uu.se/demo/ISS_Qian_et_al.html

Paper:
Qian et al
Probabilistic cell typing enables fine mapping of closely related cell types in situ.
doi.org/10.1038/s41592-019-0631-4

osmFISH mouse brain somatosensory cortex

TissUUmaps demo:
https://tissuumaps.research.it.uu.se/demo/osmFISH_Codeluppi_et_al.html

Paper:
Qian et al
Spatial organization of the somatosensory cortex revealed by osmFISH.
doi.org/10.1038/s41592-018-0175-z

Documentation

At [our website](tissuumaps.research.it.uu.se/howto.html) tissuumaps.research.it.uu.se/howto.html we have made available several video tutorials for the different uses of TissUUmaps

At our [github repository](https://github.com/wahlby-lab/TissUUmaps) https://github.com/wahlby-lab/TissUUmaps you can see the instructions and clone (get a copy) of TissUUmaps and start using it straight away

For the user of an already setup TissUUmaps viewer

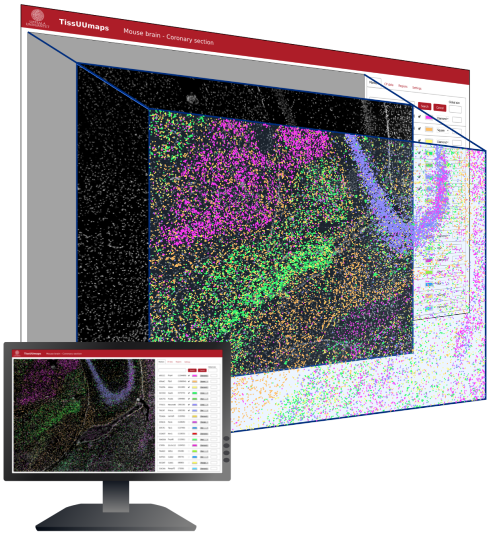

To use TissUUmaps when offered as a service already setup, image data is browsed in the main viewer to the left, it can be zoomed in and panned, and the marker data can be loaded, drawn and explored using the options in the panel on the right.

A CSV file that contains either a barcode or a gene name or both, associated to an X,Y position in the image. More data can be associated with the coordinate, all of it is available in the TissUUmaps interface.

For more information you can see the video *Viewing gene expression data* at our website https://tissuumaps.research.it.uu.se/howto.html#section-using

---

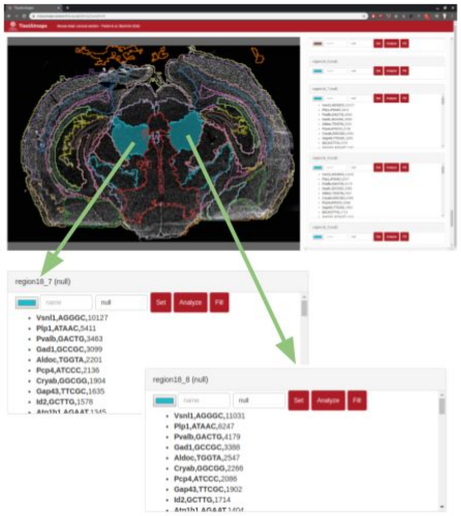

This video tutorial focuses on the creation and use of regions to explore data. Regions are a JSON file containing the polygons that describe the regions. These regions can be saved and imported. Using the TissUUmaps the data inside regions can be quantified and compared

For more information you can see the video *Using regions to explore data* at our website https://tissuumaps.research.it.uu.se/howto.html#section-using

For setting up a local TissUUmaps viewer

More detailed information on how to clone and setup your own viewer locally can be found at our github repository

Converting images to tiled formats

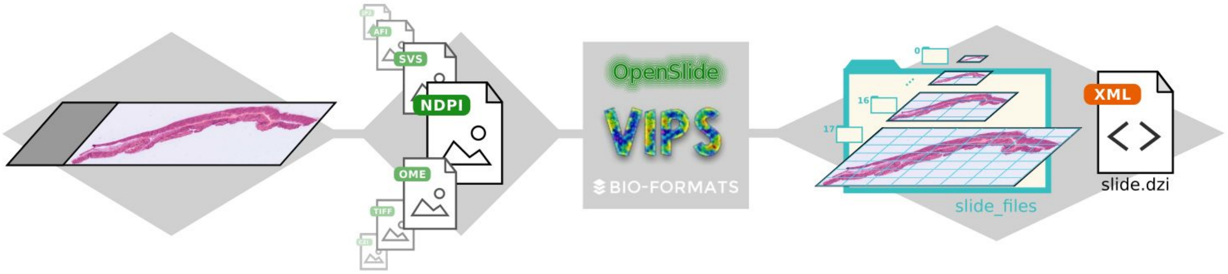

Since we don't want to use external servers to serve our images, you can convert them to an open pyramidal format where we can see individual tiles and OpenSeadragon can easily load the correct tile for you. It is called Deep Zoom Image.

This image summarizes the process of converting a whole slide image into a DZI. As long as OpenSlide can open the format, then the program VIPS will be able to convert it to a DZI.

VIPS is a super powerful image processing library. It offers a specific command to convert an image to DZI. For example:

```
vips dzsave slide.ndpi --tile-size=254 --suffix .jpg[Q=90] mySlide
```

Pointing TissUUmaps to your image data

Once you have your tiled DZI image in the computer where you will use TissUUmaps, you can clone this repository. In the HTML file example.html you just need to tell TissUUmaps where your image is. To do this, check the last script tag in the HTML and change the value of tmapp.fixed_file

```
<script>
    document.getElementById("project_title").innerText = "Project title";
    $(document).ready(function () {
        tmapp.fixed_file = "path/to/mySlide.dzi";
        tmapp.registerActions();
        tmapp.init();
    });
</script>
```

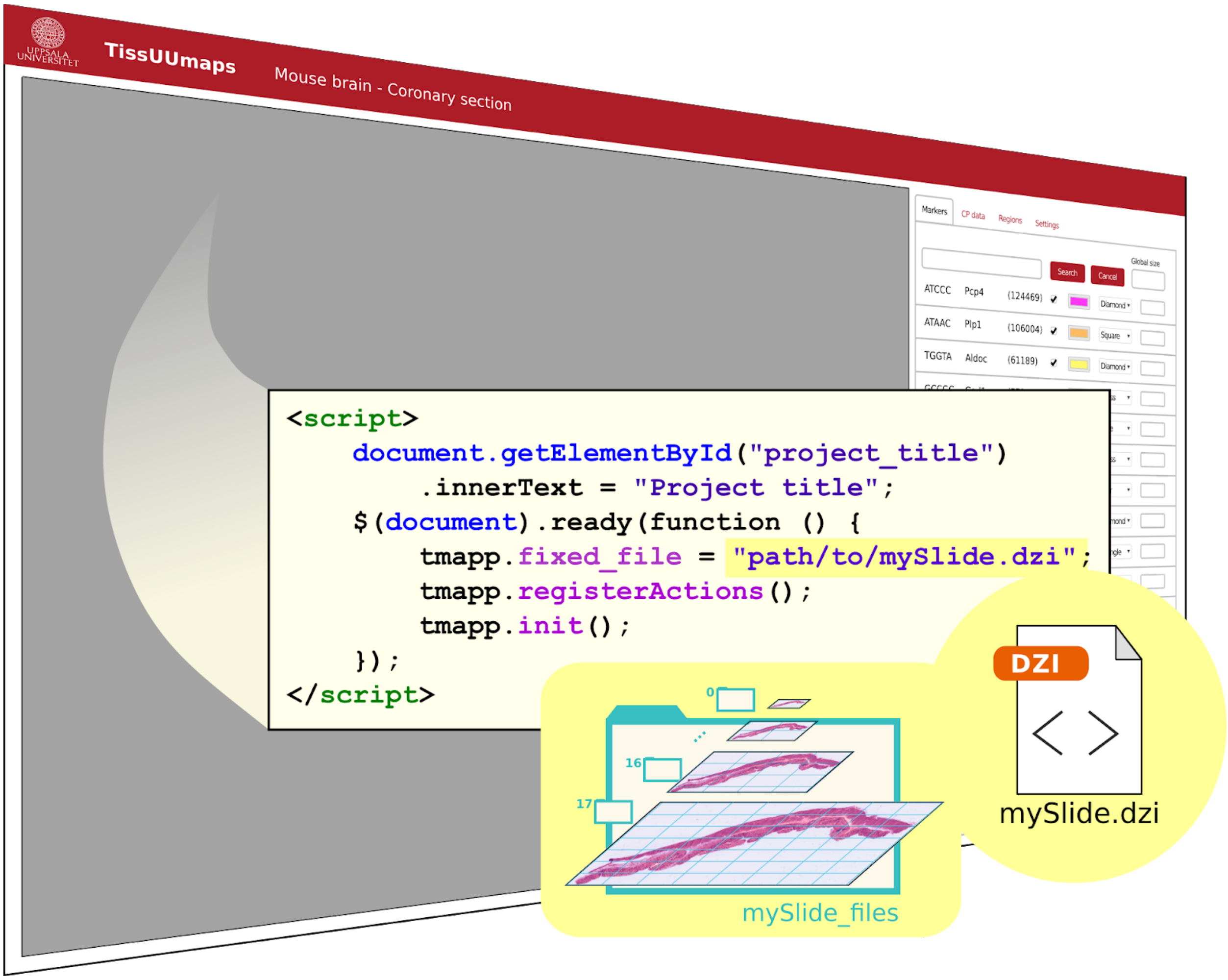

Additional information for those curious

- css
- js
- misc
- LICENSE
- README.md
- example.html
- favicon.ico
- uulogowhitetuum.png

TissUUmaps is composed of HTML and javascript files, the files that you can see in the github repo. When in your own computer, you can right-click on the example.html file and tell it to open with a browser, like chrome, firefox or safari. Your browser will use all the other files to create the TissUUmaps interface and to be able to load, process and display your data.

For setting up TissUUmaps as a service

Web applications that can be accessed from the internet are set up in servers. The servers receive requests, process them and then return to you with your web page.

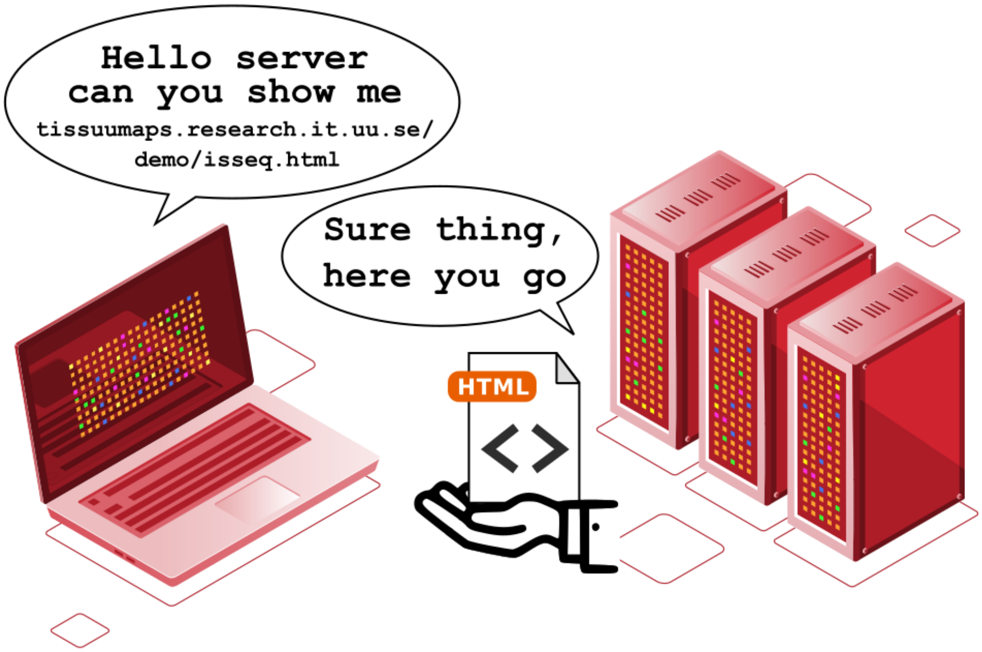

The internet as we know it runs in its majority on Apache servers. The setup of a web server is out of the scope of this documentation but we suggest the official Apache documentation at https://httpd.apache.org/docs/trunk/getting-started.html

Basically a web server is a program that takes a folder in a computer and makes it available to the internet. Once you set up a server, you just need to put the same files as in our previous section so that they can be served.

For example, our server is located at a place called `tissuumaps.research.it.uu.se` and then we want to serve you our demos, so we created a folder *demo* where we place copies of example.html that can be served to you.

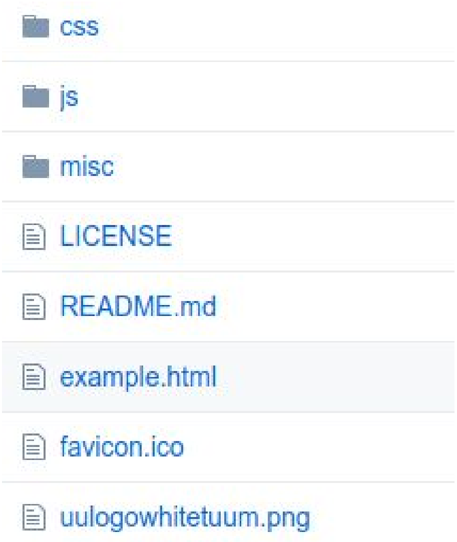

There are other servers, like NGINX and http.sever in python language that could be used for smaller scale servers, but for safety we recommend Apache.

Cloud services like Amazon Web Services offer the possibility of having your own server in the cloud so that you don’t need to set up a physical computer.

TissUUmaps API

Everything that can be done in pure HTML and javascript can be done to extend the functionality of TissUUmaps. However, to make things easier and organized. We have created special utility modules where the core functionality is programmed and additional functions can be found to interact with the interface and with the data itself. Documentation for all the modules can be found here: https://wahlby-lab.github.io/TissUUmaps/docs/

The modules are:

dataUtils	overlayUtils	regionUtils
CPDataUtils	markerUtils	HTMLElementUtils
interfaceUtils	OSDViewerUtils	

As their names suggest, `dataUtils` contains functions for dealing with common data interactions. `regionUtils` deals with functions related to regions.

In order to use the utility modules, we use the developer tools console that exist natively in browsers.

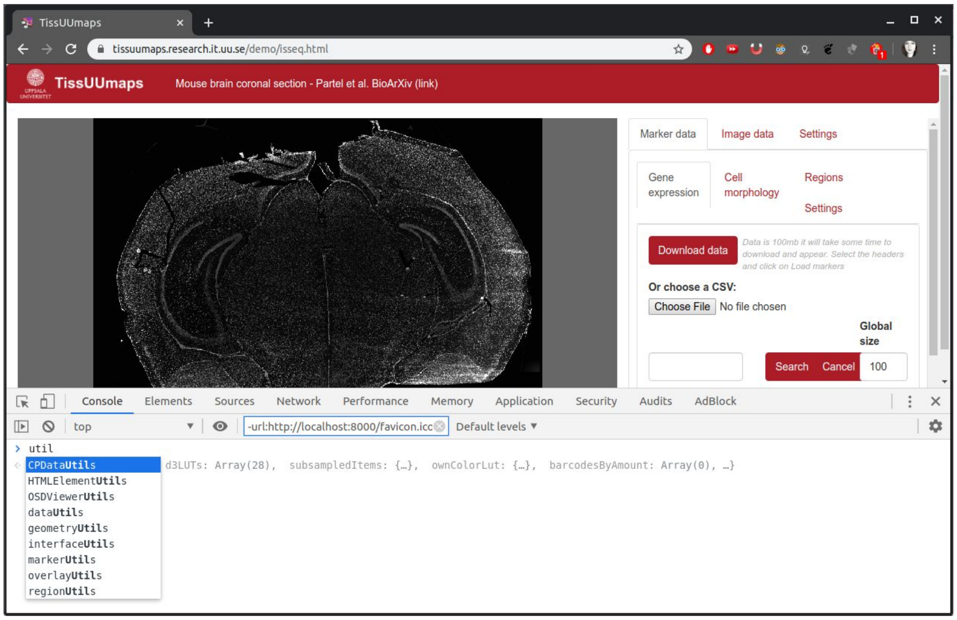

Console in Google Chrome

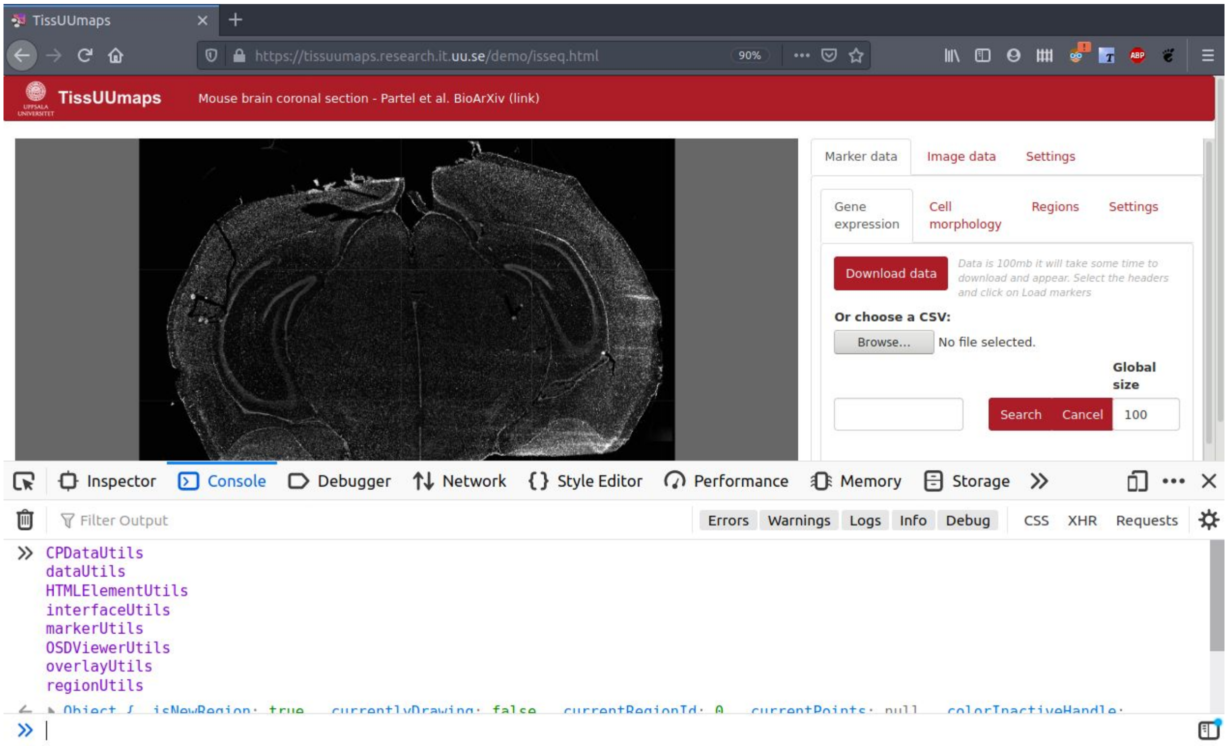

Console in Mozilla Firefox

Examples

Changing layer transparency/opacity

Everything we draw on TissUUmaps is in a layer, we refer to it as a D3 node.
The `overlayUtils` module contains all the D3 nodes that are created in the overlay

To see the available overlays you can write

`overlayUtils._d3nodes` and press enter, you will see all the existing D3 nodes.

`overlayUtils` has the function `setLayerOpacity` which asks for a name and the desired opacity ranging from 0.0 to 1. For example let's say we want all the markers to be 0.5 so that we see what is underneath. *(another way to do this is simply by changing the marker size)*

The following image shows all the nodes in the view. The node that contains the gene expression is called `ISS_markers_svgnode`. So we tell `overlayUtils` to change its opacity.

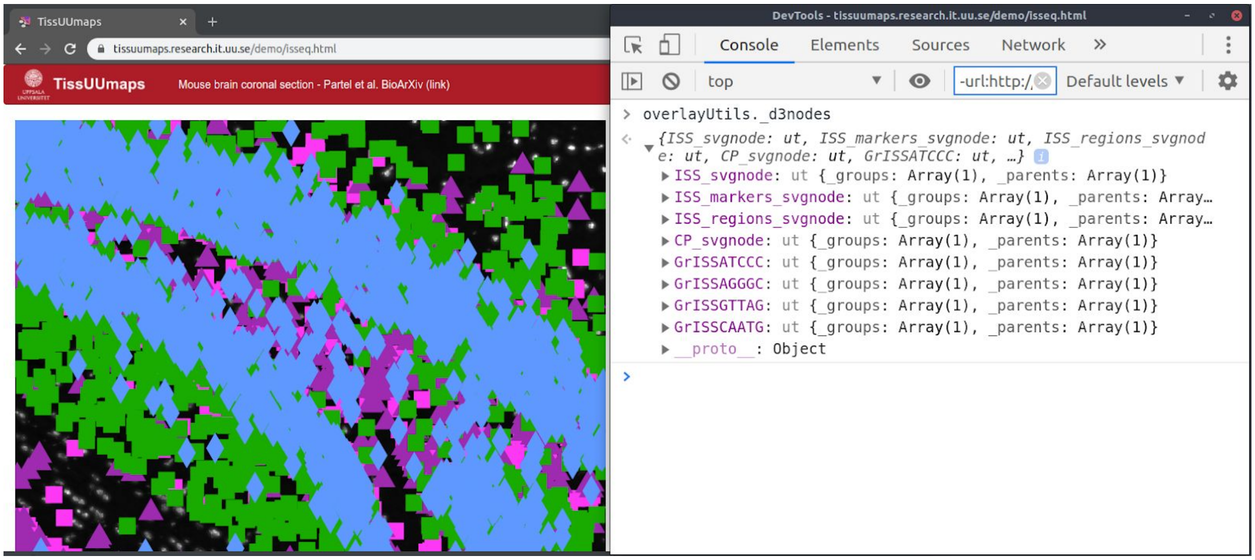

You may also notice nodes starting with the letters “Gr” these nodes contain the markers for specific genes, for example GrISSATCCC contains the markers for the gene with barcode ATCCC. If you want, you can also change the opacity of genes independently.

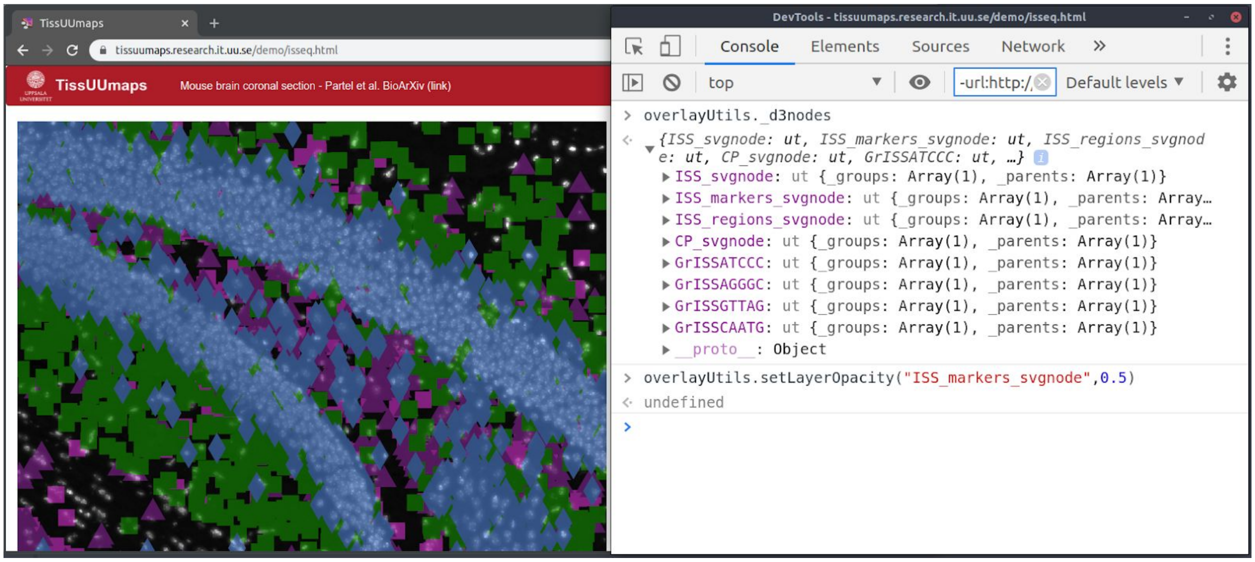

Making a histogram of the barcodes in a region

Now for a slightly more intricate example. All regions are stored in the `regionUtils` module in `regionUtils._regions` . Each region in the interface has the Analyze button which counts all the gene expression inside it. This count is stored in
`regionUtils._regions.regionName.barcodeHistogram`
Each region in the interface has a panel where the results can be observed. Every place in our interface is a place where we can place a graph made by D3. With the following code, a Histogram can be drawn inside the panel for a region, for example in this case, region1

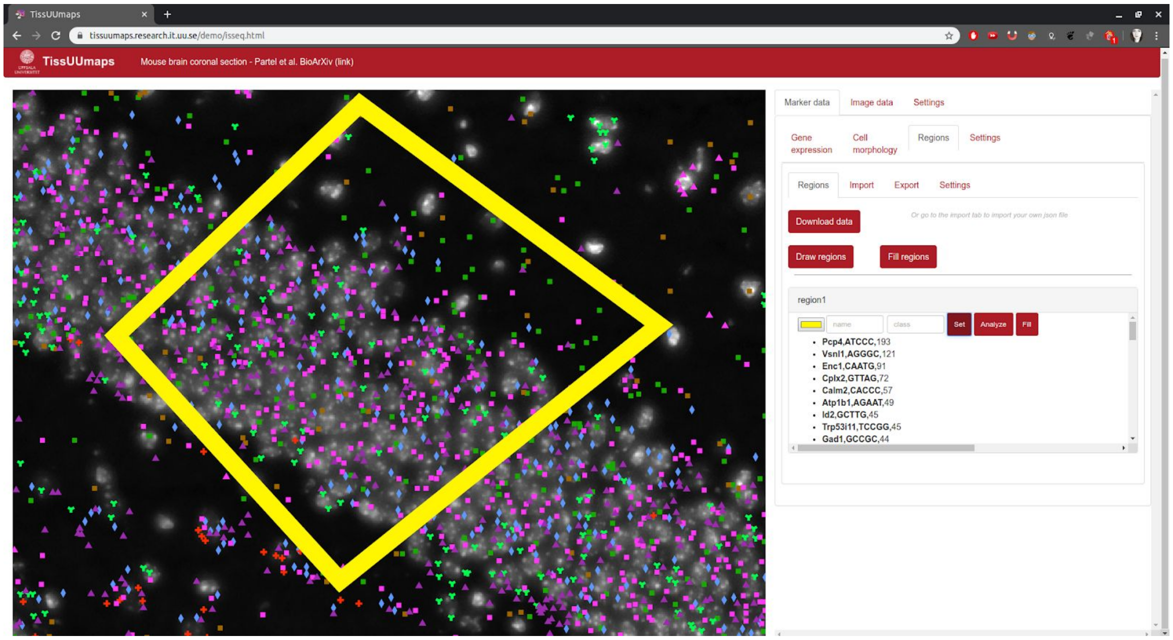

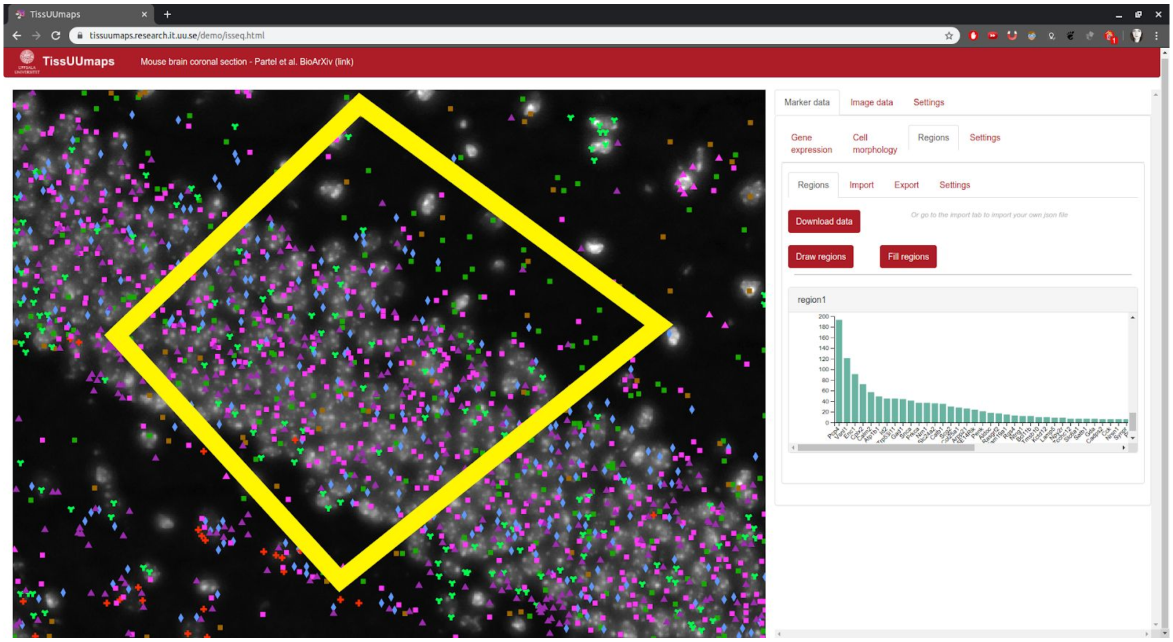

```
var data=regionUtils._regions.region1.barcodeHistogram;
var xdomain=[];
data.forEach(function(d) { xdomain.push( d.gene_name); })
var margin = {top: 30, right: 30, bottom: 70, left: 60},
   width = 1000 - margin.left - margin.right,
   height = 400 - margin.top - margin.bottom;

// append the svg object to the body of the page
var histosvg = d3.select("#region1histogram")
 .append("svg")
   .attr("width", width + margin.left + margin.right)
   .attr("height", height + margin.top + margin.bottom)
 .append("g")
   .attr("transform",
         "translate(" + margin.left + "," + margin.top + ")");

// X axis
var x = d3.scaleBand()
 .range([ 0, width ])
 .domain(xdomain)
 .padding(0.2);

histosvg.append("g")
 .attr("transform", "translate(0," + height + ")")
 .call(d3.axisBottom(x))
 .selectAll("text")
   .attr("transform", "translate(-10,0)rotate(-45)")
   .style("text-anchor", "end");

// Add Y axis
var y = d3.scaleLinear()
 .domain([0, 200])
 .range([ height, 0]);
histosvg.append("g")
 .call(d3.axisLeft(y));

// Bars
```

```
histosvg.selectAll("mybar")
 .data(data)
 .enter()
 .append("rect")
   .attr("x", function(d) { return x(d.gene_name); })
   .attr("y", function(d) { return y(d.count); })
   .attr("width", x.bandwidth())
   .attr("height", function(d) { return height - y(d.count); })
   .attr("fill", "#69b3a2")
```

More examples

We will continuously create scripts and snippets to show our users all the capabilities TissUUmaps can offer to adapt to each need. Be sure to check https://tissuumaps.research.it.uu.se/howto.html to have the latest scripting examples.
